# CD28-signaling can be partially compensated in CD28-knockout mice but is essential for virus elimination in a murine model of multiple sclerosis

**DOI:** 10.3389/fimmu.2023.1105432

**Published:** 2023-04-05

**Authors:** Kirsten Hülskötter, Fred Lühder, Eva Leitzen, Alexander Flügel, Wolfgang Baumgärtner

**Affiliations:** ^1^ Department of Pathology, University of Veterinary Medicine Hannover, Foundation, Hannover, Germany; ^2^ Institute for Neuroimmunology and Multiple Sclerosis Research (IMSF), University Medical Center Goettingen, Goettingen, Germany

**Keywords:** conventional CD28-knockout, conditional CD28-knockout, multiple sclerosis (MS) disease, Theiler’s murine encephalomyelitis virus (TMEV), immunology & infectious diseases, neuroimmunology and neuropathology

## Abstract

The intracerebral infection of mice with Theiler’s murine encephalomyelitis virus (TMEV) represents a well-established animal model for multiple sclerosis (MS). Because CD28 is the main co-stimulatory molecule for the activation of T cells, we wanted to investigate its impact on the course of the virus infection as well as on a potential development of autoimmunity as seen in susceptible mouse strains for TMEV. In the present study, 5 weeks old mice on a C57BL/6 background with conventional or tamoxifen-induced, conditional CD28-knockout were infected intracerebrally with TMEV-BeAn. In the acute phase at 14 days post TMEV-infection (dpi), both CD28-knockout strains showed virus spread within the central nervous system (CNS) as an uncommon finding in C57BL/6 mice, accompanied by histopathological changes such as reduced microglial activation. In addition, the conditional, tamoxifen-induced CD28-knockout was associated with acute clinical deterioration and weight loss, which limited the observation period for this mouse strain to 14 dpi. In the chronic phase (42 and 147 dpi) of TMEV-infection, surprisingly only 33% of conventional CD28-knockout mice showed chronic TMEV-infection with loss of motor function concomitant with increased spinal cord inflammation, characterized by T- and B cell infiltration, microglial activation and astrogliosis at 33-42 dpi. Therefore, the clinical outcome largely depends on the time point of the CD28-knockout during development of the immune system. Whereas a fatal clinical outcome can already be observed in the early phase during TMEV-infection for conditional, tamoxifen-induced CD28-knockout mice, only one third of conventional CD28-knockout mice develop clinical symptoms later, accompanied by ongoing inflammation and an inability to clear the virus. However, the development of autoimmunity could not be observed in this C57BL/6 TMEV model irrespective of the time point of CD28 deletion.

## 1 Introduction

The intracerebral inoculation with Theiler’s murine encephalomyelitis virus (TMEV) is a widely used, infectious model for neuro-inflammatory and -degenerative diseases like multiple sclerosis (MS) or epilepsy in humans (1). Susceptibility and outcome of a TMEV-infection in mice is genetically defined by the host (2). Infection of the central nervous system (CNS) is also influenced by the virus strain and as importantly, disease progression and virus spread within the nervous tissue from the cerebrum to the spinal cord is associated with changes in cell tropism (2–5). TMEV shows a strong tropism to neurons during the acute phase, which is followed by a predominant infection of glial cells and macrophages in the chronic phase of infection after three to four weeks (2–8). Briefly, the acute phase of the TMEV-infection is related to a direct virus-mediated effect predominantly on neurons and characterized by a transient encephalitis that lasts about two to three weeks after intracerebral infection in most mouse strains (1, 9). In resistant C57BL/6 mice the acute phase of TMEV-infection is restricted to the brain, especially the hippocampus, and these animals are able to eliminate the virus from the CNS within about two weeks (9). In susceptible mouse strains with an insufficient anti-viral immune response, like SJL mice, the acute phase is followed by a chronic phase, characterized by virus spread into the spinal cord and persistent TMEV-infection with concomitant development of a progressive demyelinating disease (TMEV-IDD), resembling progressive MS (1, 9). The alterations seen in the chronic phase are caused by immune-mediated tissue damage and infection of glial cells and macrophages (2–8). Thus, the division into an acute and chronic phase is not only based on the pathomorphological changes in the CNS, but also characterized by a changing cell tropism of the virus and underlying pathogenetic mechanisms (2–5).The clearance of a pathogen is critically dependent on a functioning immune system. For a virus infection, especially cytotoxic CD8^+^ T cells and NK cells are significantly involved, but the function of these cells is also embedded in the general context of a healthy immune system, involving antigen-presenting cells of the myeloid lineage as important cells for T cell activation and also T-helper cells for assisting the CD8^+^ cytotoxic response. The co-stimulatory molecule CD28 is considered the most important accessory molecule for the activation of naïve T cells (10–13). CD28 is first expressed on T cells during the β-selection in the thymus (14, 15) and keeps up its regulatory functions until the exhaustion of the T cell (16). When the CD28-mediated co-stimulatory signal is missing, T cells are unable to be fully activated and to differentiate and are becoming anergic instead. Beside its function in activation and differentiation of effector T cells, CD28 is also found to be critically involved in the maintenance of regulatory T cells (17–19). In 1993, a conventional CD28-knockout mouse was published missing the co-stimulatory molecule in all cells through all stages of life (20). These mice have some defects in germinal center reactions and antibody class switch recombination, but in principle are still able to mount cytotoxic T cell responses to certain viruses (20). Additionally, they exhibit a normal susceptibility to Leishmania major and are able to mount Th1 and Th2 responses (21). However, knockout of CD28 or blockade of CD28 with specific antibodies prevents the development of autoimmunity such as experimental autoimmune encephalitis (EAE) in rodents (22–24), and rhesus monkeys (25) or collagen-induced arthritis (26). The caveat in using conventional CD28-knockout mice is that adaptation mechanisms could have taken place where either other co-stimulatory receptors take over the function of CD28 or a different T cell repertoire with a different overall affinity/avidity of the T cell receptor (TCR) is selected in the thymus, changing the sensitivity of effector T cells for the requirement of co-stimulation during activation/differentiation. To overcome this problem, conditional CD28-knockout mice have been generated in which CD28 can be deleted pharmaceutically (27). These mice show normal expression of CD28 until administration of the inductive agent, like tamoxifen for activation of a modified estrogen receptor-coupled cre-recombinase (cre). This enables to define the exact time point of loss of CD28 expression in these mice without the problems of aberrant T cell selection in the thymus.

For human autoimmune diseases, aberrations of CD28-expression appears to influence the risk for MS relapses (28) as well as severity of Crohn’s disease (29). In addition, it was hypothesized that if a chronic virus infection of the CNS is involved in the disease pathology, as recently proposed for MS (30, 31), a downregulation of the immune response might be disadvantageous for the host. However, beside the “molecular mimicry” hypothesis (32) for the involvement of pathogens in the pathogenesis of autoimmune diseases, it is currently unknown how pathogens could contribute to their development.

In the present study we investigated how mice with a constitutive (CD28 ^–/–^; further named CD28KO) or conditional deletion of CD28 (CD28^-/flox^ Cre^+/-^; further named Cre) are able to deal with a TMEV-infection in comparison to controls without Cre-expression (CD28^-/flox^ Cre^-/-^; further named B6) and to controls without tamoxifen administration. It could be shown that the CD28 knockout renders genetically resistant mice susceptible for chronic TMEV-infection reflected by persistent virus presence, modified immune responses and histopathological changes within the CNS.

## 2 Materials and methods

### 2.1 Mice and housing

Conditional CD28-knockout mice with cre-expression (CD28^-/flox^ Cre^+/-^; Cre) and without cre-expression (CD28^-/flox^ Cre^-/-^; B6) (27) as well mice with a conventional CD28 knockout (CD28^-/-^; CD28KO) (20) on a C57BL/6 background were bred at the central animal facility of the Institute for Multiple Sclerosis Research (IMSF), University of Göttingen. The study took place in the Department for Pathology of the University of Veterinary Medicine Hannover, where the mice were housed in individual ventilated cages (IVC) with ad libitum access to water and food (ssniff Spezialdiäten GmbH, DE- 59494 Soest, cat. V1534-000). All experiments were performed in accordance with German law and approved by the Lower Saxony State Office for Consumer Protection and Food Safety as the responsible authority (Niedersächsisches Landesamt für Verbraucherschutz- und Lebensmittelsicherheit (LAVES), Oldenburg, Germany; permission number: -17/2418).

### 2.2 Induction of CD28-knockout

The knockout of CD28 was induced in Cre-expressing FEC2/FLCK CD28KO mice (CD28^-/flox^ Cre^+/-^) by three oral gavages of tamoxifen (3 mg tamoxifen/100µl rapeseed oil) at 5, 7 and 9 dpi, using a flexible 20 G feeding tube (Instech, cat. FTP-20-38-50) as described before (4).

### 2.3 TMEV-infection

All mice of the study were infected intracerebrally at the age of five weeks with 20µl of a TMEV-BeAn-1-TiHo cell culture supernatant of 2.7x10^7^ plaque-forming units (PFU)/ml (infective dose of 5.4x10^5^ PFU) under injection-anesthesia as described before (33).

### 2.4 Study design

#### 2.4.1 Acute phase of infection

To study the acute phase of TMEV-infection, mice with conventional innate (CD28KO) and tamoxifen-induced, conditional knockout (Cre-Tam) of CD28 were infected intracerebrally at the age of five weeks. The conditional knockout was induced 5 days post infection (dpi) in Cre-Tam mice by oral gavages of tamoxifen at 5, 7 and 9 dpi. Clinical scoring and motor tests (RotaRod) were performed weekly. The mice were sacrificed at 14 dpi. Control animals without CD28 knockout were treated accordingly.

As controls for the wild type-phenotype mice with loxP-flanked exons 2 and 3 of CD28 (flox) without expression of cre-recombinase (CD28^-/flox^ Cre^-/-^, further named: B6) were used (27). Additionally, CD28^-/flox^ Cre^-/-^ animals without tamoxifen application (nT) were involved in this study, since tamoxifen is known to influence the immune response after TMEV-infection (4, 34).

The genotypes, number of animals and information about infection, Tam application and necropsy are presented in **Table 1**.

**Table 1:** Overview of conditional and conventional CD28-knockout mice investigated in the early phase of TMEV-infection at 14 dpi, including the group names, genetic background, age at TMEV-infection, days of oral tamoxifen application, number of animals, information about CD28 knockout status and/or induction, day of necropsy after TMEV infection.

Group name	Genetic background	Age at TMEV infection	Tamoxifen application (dpi)	Number of animals	CD28 KO	Necropsy (dpi)
B6-nT	CD28^-/flox^ Cre^-/-^	5 weeks	None	6	No	14 dpi
B6-Tam	CD28^-/flox^ Cre^-/-^	5 weeks	5, 7 and 9 dpi	10	No	14 dpi
CD28KO	CD28 ^-/-^	5 weeks	None	6	Yes	14 dpi
Cre-Tam	CD28^-/flox^ Cre^+/-^	5 weeks	5, 7 and 9 dpi	17	Yes	14 dpi

B6, mice without Cre expression (conditional CD28-knockout cannot be induced by tamoxifen); Cre, mice with cre-recombinase expression (conditional CD28-knockout can be induced by tamoxifen); CD28KO, mice with conventional; innate CD28-knockout; nT, mice without Cre expression and without tamoxifen application; Tam, oral tamoxifen application 5; 7 and 9 days post infection; TMEV, Theiler’s murine encephalomyelitis virus; dpi, days post TMEV infection.

#### 2.4.2 Chronic phase of infection

For investigations on the chronic TMEV-infection, only mice with conventional innate CD28-knockout (CD28KO) were used. Due to detrimental effects of the induced conditional CD28-knockout on the clinical score of the Cre-Tam mice, these animals were not included in the long-term studies for animal welfare reasons. Of the mice with conventional CD28-knockout (CD28KO) planned for necropsy at 42 dpi (n=6) and 147 dpi (n=6, data not shown), one third (n= 4 out of 12) developed motoric deficiencies starting at 21-33 dpi. These four mice were thus evaluated separately as ‘responders’ (CD28KO-R). Two of the four CD28KO-R mice developed hind limb paresis and were immediately euthanized for humane reasons at 33 (n=1) or 35 dpi (n=1), respectively. Two of the four CD28KO-R mice showed only mild to moderate clinical signs and thus reached the scheduled necropsy date at 42 dpi (n=2). The CD28KO-R group was thus composed of n=4 animals euthanized at 33 (n=1), 35 (n=1) and 42 dpi (n=2). These were compared to the CD28KO mice without clinical signs (n=4) and controls without tamoxifen application (B6-nT, n=6), all sacrificed at 42 dpi. Remaining CD28KO mice that did not show clinical signs until 42 dpi (n=4) as well as further B6-nT control mice (n=7) did not develop any clinical signs until 147 dpi (endpoint of investigation). At 147 dpi, there was no difference in clinical as well as histological data between these animals (data not shown). The genotypes, number of animals and information about infection, Tam application and necropsy are presented in **Table 2**.

**Table 2:** Overview of conventional CD28-knockout mice in the investigation on the chronic phase of TMEV-infection at 33-42 dpi, including the group names, genetic background, age at TMEV-infection, days of oral tamoxifen application, number of animals, information about CD28 knockout status, day of necropsy after TMEV infection.

Group name	Genetic background	Age at TMEV infection	Tamoxifen application	Number of animals	CD28 KO	Necropsy (dpi)
B6-nT	CD28^-/flox^ Cre^-/-^	5 weeks	None	6	No	42 dpi
CD28KO	CD28 ^-/-^	5 weeks	None	4	Yes	42 dpi
CD28KO-R	CD28 ^-/-^	5 weeks	None	4	Yes	33,35 and 42 dpi
B6-nT	CD28^-/flox^ Cre^-/-^	5 weeks	None	7	No	147 dpi
CD28KO	CD28 ^-/-^	5 weeks	None	4	Yes	147 dpi

B6-nT, mice without Cre expression and without tamoxifen application; CD28KO, mice with conventional innate CD28-knockout without motoric deficiencies; CD28KO-R, mice with conventional innate CD28-knockout and motor deficiencies (responders); TMEV, Theiler’s murine encephalomyelitis virus; wpp. weeks post-partum; dpi, days post TMEV infection.

### 2.5 Clinical scoring, RotaRod^®^

Mice were visually inspected daily; clinical scoring (appearance [0-3], activity [0-3], gait [0-4]) and RotaRod^®^-testing for motor skills were performed weekly as described (4). The intervals of clinical scoring were increased according to the animals’ clinical signs as described before (4).

### 2.6 Necropsy

Animals were sacrificed at 14 (n=39), 42 (n=12) or 147 (n= 11, data not shown) days post infection (dpi). Mice with CD28 knockout that reached the pre-defined termination criteria (weight loss ≥ 20%, high clinical scores, paralysis of limbs) were euthanized for humane reasons at 14 (Cre-Tam), 33 (n=1) and 35 (n=1) dpi (CD28KO-R). After euthanasia, mice were perfused with phosphate buffered saline (PBS) at a flow rate of 3.75 ml/min through the left ventricle. Brain, spinal cord, thymus, spleen, and intestine were removed and fixed in 4% formaldehyde solution followed by paraffin embedding.

### 2.7 Histology and immunohistochemistry – staining and scoring

For histology and immunohistochemistry, the formalin-fixed, paraffin-embedded (FFPE) tissue was cut to 2 µm thick sections and mounted on glass slides (Thermo Scientific, Superfrost^®^ Plus, cat. J1800AMNZ). Hematoxylin-eosin (HE) stained sagittal sections of the brain were divided into 10 regions (as seen in **Supplementary Figure S1**) and scores (0-3) for hyper-cellularity, perivascular infiltration, meningeal infiltration, vacuolization, and neuronal/axonal damage (4, 35) were added up. The scores (0-3) for hyper-cellularity in the white matter, the grey matter, perivascular infiltration, vacuolization and meningeal infiltration from three spinal cord segments (cervical, thoracic, and lumbar) each divided into 6 regions (left/right, dorsal, middle and ventral) were added up for every animal.

Immunohistochemistry was performed applying the avidin-biotin complex (ABC)-method with 3,3’-diaminobenzidine (DAB)-labelling as described before (36). The pretreatment and concentrations of primary antibodies are presented in **Table 3** below:

**Table 3:** List of antibodies for immunohistochemistry.

Abbre-viation	Primary antibody	Company, #cat-no.	Concentration of primary antibody	Pretreatment	Blocking	Secondary antibody (1:200)	Company, cat-no#2
Beta-APP	Anti-APP A4 Mouse mAb, clone 22C11	Merck, #MAB348	1:2000	20 min. microwave, citrate buffer (pH 6)	Goat-serum	Biotinylated-goat-anti-mouse IgG	Vector, #BA9200
Caspase 3	Anti-cleaved caspase 3 (Asp175) Rabbit pAb	Cell Signaling, #9661	1:200	20 min. microwave, citrate buffer (pH 6)	Goat-serum	Biotinylated-goat-anti-rabbit IgG	Vector, #BA1000
CD3	Anti-human CD3 Rabbit pAb	Dako, #A0452	1:250	20 min. microwave, citrate buffer (pH 6)	Goat-serum	Biotinylated-goat-anti-rabbit IgG	Vector, #BA1000
CD45R	Biotinylated-anti mouse CD45R/B220 Rat mAb	BD Pharmin-gen, #553085	1:1000	20 min. microwave, citrate buffer (pH 6)	None	None	None
GFAP	Anti-cow GFAP Rabbit pAb	Agilent, #Z033429-2	1:1000	None	Goat-serum	Biotinylated-goat-anti-rabbit IgG	Vector, #BA1000
Iba-1	Anti-Iba1 Rabbit pAb	Wako, #019-19741	1:500	None	Goat-serum	Biotinylated-goat-anti-rabbit IgG	Vector, #BA1000
S100A10	Anti- S100A10 Rabbit mAb	Bio Techne, #Ab JF0987	1:100	None	Goat-serum	Biotinylated-goat-anti-rabbit IgG	Vector, #BA1000
TCF-1	Anti-TCF1/TCF7 (CD63D9) Rabbit mAb	Cell Signaling, #2203	1:1000	20 min. microwave, citrate buffer (pH 6)	Goat-serum +1% BSA +0,1% Triton X	Biotinylated-goat-anti-rabbit IgG	Vector, #BA1000
TMEV	Anti-TMEV Rabbit pAb	(30)	1:2000	None	Goat-serum	Biotinylated-goat-anti-rabbit IgG	Vector, #BA1000

APP, amyloid precursor protein (marker for axonal damage); caspase 3 (marker for apoptotic cells), CD3 (T cell marker), CD45R (B cell marker), GFAP, glial fibrillary acidic protein (astrocyte marker); Iba-1, ionized calcium-binding adapter molecule 1 (marker for macrophages and microglia); S100A10 (marker for neurotrophic astrocytes); TCF, T cell factor (naïve T cell marker); TMEV, Theiler’s murine encephalomyelitis virus; mAb, monoclonal antibody; pAb, polyclonal antibody.

For morphometry and figures, stained sections were scanned with extended focal image (EFI) on an Olympus Slide Scanner VS200. Tissue detection was done by thresholding (CellSens, ImageJ). Positive stained areas and cell counts were obtained according to the requirements by manual counting on glass slides (TMEV, CD45R, Caspase 3, beta amyloid precursor protein [beta-APP], S100A10) or applying a neural network (CD3 [Olympus cellSens imaging software VS200]) trained on respective tissue to detect the cell numbers per area. Brain and spinal cord sections with glial markers (ionized calcium-binding adapter molecule 1 [Iba-1] and glial fibrillary acidic protein [GFAP]) were digitalized (VS200) followed by thresholding and automatic detection of the percentage of positive areas (ImageJ, QuPath) to account for changes in cell volumes during the inflammatory response. The cell numbers and positive areas were evaluated on one sagittal brain section (including all 10 regions as defined above) or three spinal cord sections (cervical, thoracic, lumbar) combined into one evaluated area, respectively.

### 2.8 Figures, graphs and statistics

Photographs for the figures were obtained from scanned slides (VS200) and edited with GIMP 2.10.32 (GNU Image Manipulation Program). Graphs were created with Graphpad Prism (Graphpad Software Inc.) and show scatter plots (one dot per animal) with median (black bar) and 95% confidence intervals (black error bars). Statistical analyses were conducted using SPSS for Windows TM v. 27 (IBM^®^ SPSS^®^ Statistics, SPSS Inc., Chicago, IL, United States). Data were analyzed for normal distribution using the Shapiro–Wilk test. Significant differences between the groups were investigated *via* Kruskal–Wallis tests followed by Dunn–Bonferroni *post hoc* testing with correction of significance levels for multiple testing. Corrected p-values of ≤ 0.05 were recognized as statistically significant and indicated by an asterisk (*) within the graphs. Figures of multiple panels were generated with GIMP and Inkscape 1.2 (Inkscape Community).

## 3 Results

### 3.1 Conditional and conventional CD28-knockout mice are unable to eliminate the virus during the acute phase (14 dpi) of a TMEV-infection

**Figure 1:**
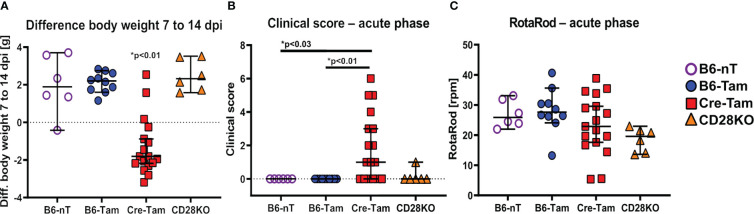
**(A-C)** Assessment of general parameters and clinical score during the acute phase of a Theiler’s murine encephalomyelitis virus (TMEV)-infection at 14 days post infection (dpi). **(A)** Mice with tamoxifen-induced CD28-knockout (Cre-Tam, n=17) show a significant loss of the body weight within the second week of infection (7 to 14 dpi). **(B)** At 14 dpi, the clinical score of Cre-Tam mice (n=17) is significantly increased compared to B6-nT (n=6) and B6-Tam (n=10) controls. **(C)** RotaRod-performance was similar and lacked significant differences between the groups at 14 dpi. Graphs show scatter plots (one dot per animal) with median (black bar) and 95% confidence intervals (black error bars). Statistically significant p-values are depicted within the graphs (Kruskal-Wallis test followed by Dunn-Bonferroni *post-hoc* test and significance level correction for multiple testing).

Mice with tamoxifen-induced, conditional knockout of CD28 (Cre-Tam) showed a marked loss of body weight between 7 and 14 dpi (**Figure 1A**). Additionally, only Cre-Tam mice showed significantly increased clinical scores (**Figure 1B**) but without measurable effects on their RotaRod performance (**Figure 1C**) at 14 dpi.

**Figure 2:**
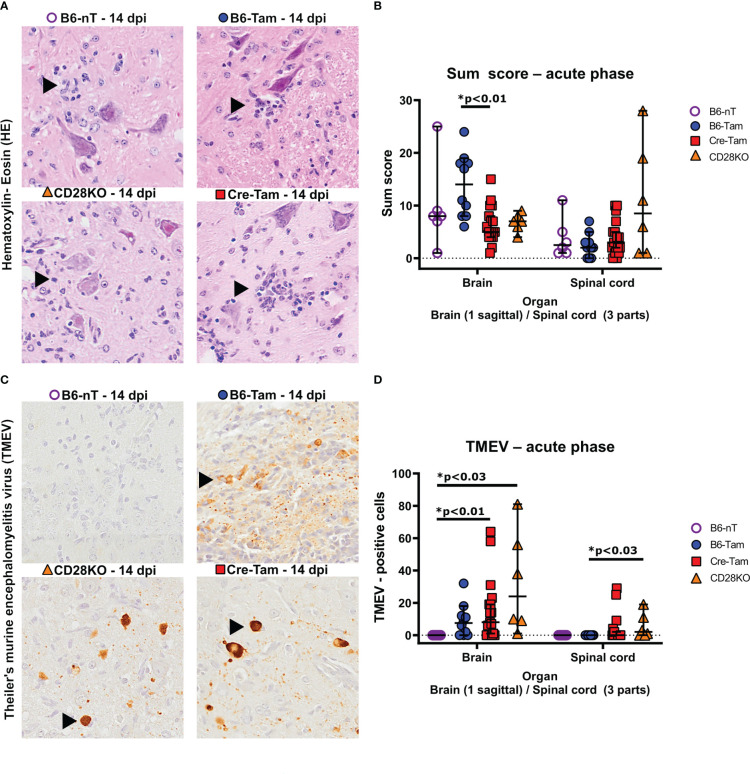
**(A, B)** Semi-quantitative scores of the brains and spinal cords of TMEV-infected B6-nT mice (no CD28-knockout, no tamoxifen application, n=6), B6-Tam mice (no CD28-knockout, tamoxifen application, n=10), CD28KO mice (CD28-knockout, no tamoxifen application, n=6) and Cre-Tam mice (CD28-knockout, tamoxifen application, n=17) using hematoxylin-eosin (HE) stained sections in the acute phase at 14 days post infection (dpi). **(A)** Representative pictures showing inflammatory infiltrates (arrowheads) within the lumbar spinal cords in Theiler’s murine encephalomyelitis virus (TMEV) infected mice at 14 dpi. **(B)** Summarized graphical presentation of semi-quantitative scores obtained in 10 brain regions and 6 spinal cord regions at 14 dpi, showing significantly lower scores within the brains of CD28-knockout (Cre-Tam) mice compared to B6-Tam controls and similar scores within the spinal cord. **(C, D)** Immunohistochemical detection of TMEV-antigen within the brains and spinal cords of TMEV-infected mice at 14 dpi. **(C)** Representative pictures showing no TMEV-antigen in B6-nT mice (upper left) and persisting TMEV-antigen within the hippocampus (B6-Tam, upper right) and cerebral cortex (CD28KO, lower left; Cre-Tam, lower right) at 14 dpi. **(D)** In contrast to B6-nT-control animals, mice with tamoxifen application (B6-Tam) as well as mice with CD28-knockout (Cre-Tam, CD28KO) showed virus antigen in the brain at 14 dpi. Virus spread into the spinal cord was only detected in mice with CD28-knockout (Cre-Tam, CD28KO) and reached statistically significance between CD28KO mice and B6-Tam controls. Graphs show scatter plots (one dot per animal) with median (black bar) and 95% confidence intervals (black error bars). Statistically significant p-values are depicted within the graphs (Kruskal-Wallis test followed by Dunn-Bonferroni *post-hoc* test and significance level correction for multiple testing).

Histopathological examination of the brain and spinal cord revealed that the sum score in HE-stained brain sections is significantly lower in Cre-Tam mice compared to the B6-Tam controls without CD28 deletion (**Figures 2A, B**). Inflammation within the spinal cord was most apparent within the lumbar region of all mouse strains (**Figure 2A**). Concerning the virus clearance, there were also differences between the groups. Whereas B6 mice without tamoxifen administration (B6-nT) completely cleared the virus by 14 dpi as expected (**Figure 2C**, upper left), mice with tamoxifen application (B6-Tam) still show virus antigen within the brain at 14 dpi (**Figure 2C**, upper right), as reported before (28). Virus persistence was also seen in both mouse strains in which CD28 had been deleted (**Figures 2C, D**). Remaining TMEV-antigen was detected preferably within the hippocampus of mice without CD28 knockout but tamoxifen application (B6-Tam), while in both mouse strains with CD28-knockout (CD28KO, Cre-Tam), the antigen was more likely to be spread over more brain regions, except for the olfactory bulb, forebrain, and cerebellum (**Supplementary Figure S1**). Highest numbers of TMEV-antigen positive cells were detected within the cerebral cortex, hippocampus and thalamus of both CD28-knockout strains (**Supplementary Figure S1**). Furthermore, only in mice with CD28-knockout (Cre-Tam, CD28KO), an unexpected virus spread into the spinal cord was seen (**Figure 2D**).

**Figure 3:**
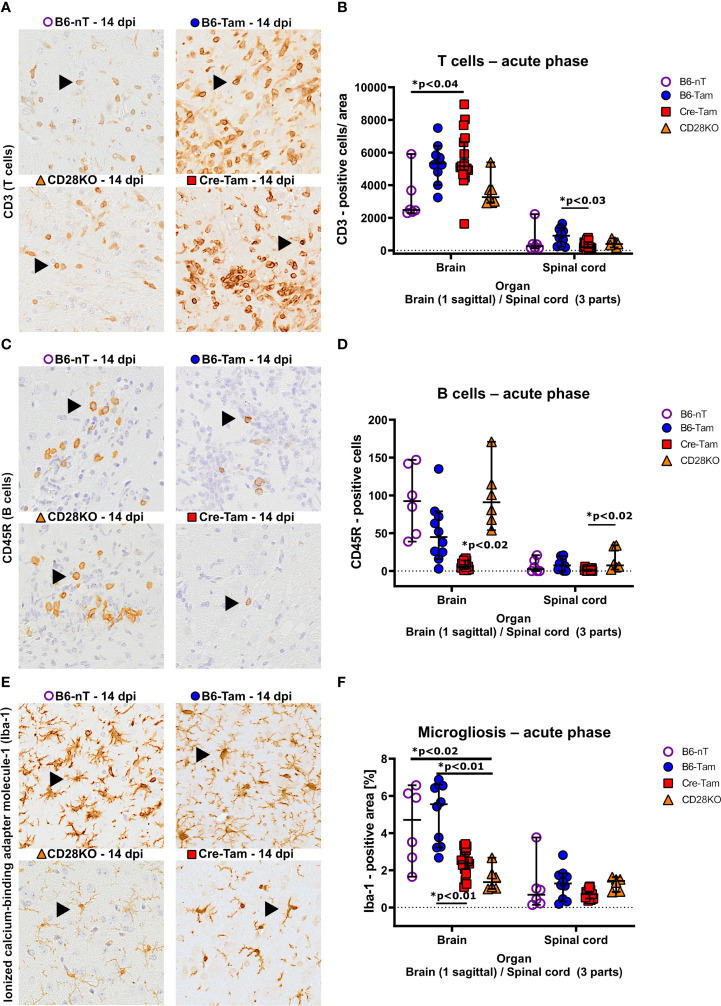
**(A, B)** Immunohistochemical detection of CD3-positive T cell infiltration into the brain and spinal cord of Theiler’s murine encephalomyelitis virus (TMEV)-infected B6-nT mice (no CD28-knockout, no tamoxifen application, n=6), B6-Tam mice (no CD28-knockout, tamoxifen application, n=10), CD28KO mice (CD28-knockout, no tamoxifen application, n=6) and Cre-Tam mice (CD28-knockout, tamoxifen application, n=17) in the acute phase at 14 days post infection (dpi). **(A)** Representative pictures showing moderate T cell infiltration (arrowheads) into the thalamus of B6-nT (upper left) and CD28KO mice (lower left) in contrast to high numbers of infiltrating T cells in B6-Tam (upper right) and Cre-Tam mice (lower right) at 14 dpi. **(B)** Quantitative evaluation of T cells per area (0.5 cm² brain, 0.07cm² spinal cord) reveals increased infiltration into the brain of Cre-Tam compared to B6-nT controls and spinal cord of B6-Tam mice, compared to Cre-Tam mice. **(C, D)** Infiltration of CD45R-positive B cells into the brain and spinal cord of TMEV-infected mice at 14 dpi. **(C)** Representative pictures showing moderate infiltration of B cells (arrowheads) into the hippocampus of TMEV-infected B6-nT (upper left) and CD28KO mice (lower left) in contrast to low numbers of infiltrating B cells in B6-Tam (upper right) and Cre-Tam mice (lower right) at 14 dpi. **(C)** Quantitative analysis shows significantly decreased B cell infiltration in the brain of Cre-Tam mice compared to all other groups. Within the spinal cord, B cell numbers are significantly lower in Cre-Tam mice, compared to CD28KO mice at 14 dpi. **(E, F)** Analysis of microgliosis within the brain and spinal cord of TMEV-infected mice at 14 dpi. **(E)** Representative pictures of activated, ionized calcium-binding adapter molecule-1 (Iba-1)-positive microglia/macrophages within the thalamus, showing increased cell numbers and ramification in B6-nT (upper left) and B6-Tam mice (upper right) in contrast to lower cell numbers with only few cell processes in CD28KO (lower left) and Cre-Tam mice (lower right) at 14 dpi. **(F)** A detailed analysis of Iba-1 positive areas reveals significantly decreased microgliosis within the brain of CD28-knockout mice (Cre-Tam, CD28KO) compared to controls (B6-nT, B6-Tam) at 14 dpi. Within the spinal cord, there is no difference at 14 dpi concerning microgliosis. Graphs show scatter plots (one dot per animal) with median (black bar) and 95% confidence intervals (black error bars). Statistically significant p-values are depicted within the graphs (Kruskal-Wallis test followed by Dunn-Bonferroni *post-hoc* test and significance level correction for multiple testing).

Immunohistochemical assessment of the local immune response by counting infiltrating immune cells into the CNS revealed significant differences in the T- and B cell response as well as microglial activation. It was observed that mice with conditional, tamoxifen-induced CD28-knockout mice (Cre-Tam, **Figure 3A**, lower right) showed increased T cell infiltration into the brain (**Figure 3B**). The T cell infiltration in B6-nT mice without tamoxifen application (**Figure 3A**, upper left) and animals with conventional CD28-knockout (**Figure 3A**, lower left) were at similarly low levels, despite the presence of TMEV-antigen in the CNS in the CD28KO mice. Within the spinal cord, there were no significant differences in T cell infiltration between the groups at 14 dpi (**Figure 3B**). The number of B cells in B6-nT mice without tamoxifen application (**Figure 3C**, upper left) and mice with conventional CD28-knockout (**Figure 3C**, lower left) were, as seen for T cell infiltration, at comparable levels (**Figure 3D**). Contrary to T cells, the B cell infiltration showed the opposite trend with non-significantly reduced B cell numbers in tamoxifen-treated B6-Tam mice (**Figure 3C**, upper right) and significantly reduced B cell numbers in Cre-Tam mice with conditional CD28 knockout (**Figure 3C**, lower right) in both the brain and the spinal cord (**Figure 3D**). Prominent reactive microgliosis was seen in the brain of B6-nT and B6-Tam mice without CD28-knockout (**Figure 3E**, upper left and upper right). Within the brain, microglial activation was significantly reduced in Cre-Tam and CD28KO mice with knockout of CD28 (**Figures 3E, F**). Within the spinal cord, there was no significant difference between mice with or without CD28-knockout (**Figure 3F**).

The number of apoptotic cells (caspase 3 positive cells) within the brain and spinal cord was not elevated in CD28-knockout mice compared to the controls at 14 dpi (**Supplementary Figure S2**). In addition, there was no increase in axonal damage (beta APP positive axons) within the spinal cord of CD28-knockout mice in the early phase of TMEV-infection at 14 dpi (**Supplementary Figure S2**).

The summarized results of the clinical and histopathological findings in intracerebrally TMEV-infected mice reveal that mice lacking CD28-expression (Cre-Tam, CD28KO) show virus persistence and spreading of virus within the brain as well as into the spinal cord at 14 dpi. This is correlated with significantly less microglial activation, despite presence of virus antigen within the CNS. T- and B cell infiltration seem to be influenced by tamoxifen administration and CD28-knockout in a mutually enhancing manner. The influence of tamoxifen on the number of infiltrating lymphocytes can be seen in the direct comparison with B6-nT controls, but only reaches statistical significance in Cre-Tam mice with CD28-knockout, that show significantly increased T cell infiltration and decreased B-cell infiltration.

### 3.2 Conventional CD28-knockout mice partially develop a clinical phenotype during the chronic phase (21-42 dpi) of a TMEV-infection

**Figure 4:**
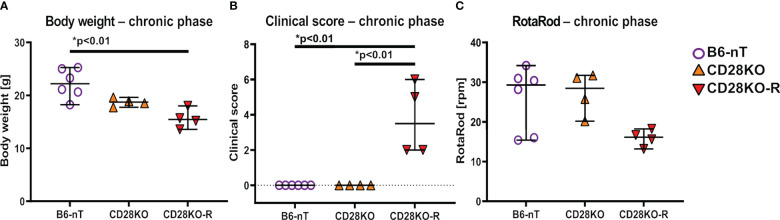
**(A–C)** Assessment of general parameters and clinical score during the chronic phase of Theiler’s murine encephalomyelitis virus (TMEV)-infection. TMEV-infected conventional CD28-knockout mice with clinical signs in the chronic phase, termed ‘responder CD28KO’ mice, (CD28KO-R, n=4) were euthanized at 33 (n=1), 35 (n=1) and 42 dpi (n=2) and compared to CD28-knockout mice without clinical signs (CD28KO, n=4) and control mice without knockout (B6-nT, n= 6) that were sacrificed on 42 dpi. **(A)** CD28KO-R mice show a significantly lower body weight at the end of experiment, compared to B6-nT controls. **(B)** B6-nT and CD28KO mice do not show a clinical score at 42 dpi. The clinical scores of CD28KO-R mice (n=4) are elevated significantly at the end of experiment at 33, 35 and 42 dpi, respectively. **(C)** RotaRod performance tests show a mildly reduced performance of CD28KO-R mice compared to CD28KO mice, that however reaches no statistical significance (p= 0.104). Graphs show scatter plots (one dot per animal) with median (black bar) and 95% confidence intervals (black error bars). Statistically significant p-values are depicted within the graphs (Kruskal-Wallis test followed by Dunn-Bonferroni *post-hoc* test and significance level correction for multiple testing).

Because TMEV has not been cleared yet at 14 dpi, it was interesting to follow up the fate of the mice also in the chronic stage of TMEV infection. In susceptible SJL mice, the chronic phase is characterized by progressive clinical signs and loss of myelin caused by different immunopathologies of this model (1). Since the CD28KO mice were unable to clear the virus within 2 weeks after infection, a longer period was studied to see whether CD28-deficiency results in a similar fate on the otherwise resistant C57BL/6 background. Due to the detrimental effects of the tamoxifen-induced conditional CD28-knockout in the early phase of TMEV-infection (weight loss, clinical signs), Cre-Tam mice were not included in this part of the study for animal welfare reasons. Mice with a conventional CD28-knockout (CD28KO) either showed no clinical signs, similar to the control animals (B6-nT) or developed motoric deficiencies at 21 (n=1), 32 (n=1) and 33 (n=2) days post TMEV-infection. Mice that developed these clinical signs were thus evaluated separately as ‘responders’ (CD28KO-R, n= 4 out of 12). At the end of experiment, the four responders with conventional CD28-knockout had a lower body weight (**Figure 4A**) and showed increased clinical scores (**Figure 4B**). The high clinical scores in CD28KO-R mice were mainly determined by hind limb paresis (score 4) of two mice at 33 (n=1) and 35 dpi (n=1). Mice with hind limb paresis were euthanized immediately for humane reasons and thus not put on the RotaRod. Accordingly, the presented data (**Figure 4C**) show the last RotaRod performance on 28 dpi of mice prior to the hind limb paresis and 42 dpi of the mice with mild gait abnormalities. It can be seen, that anyways, the motor skills of the CD28KO-R mice are non-significantly reduced compared to the non-responder CD28KO mice (**Figure 4C**).

**Figure 5:**
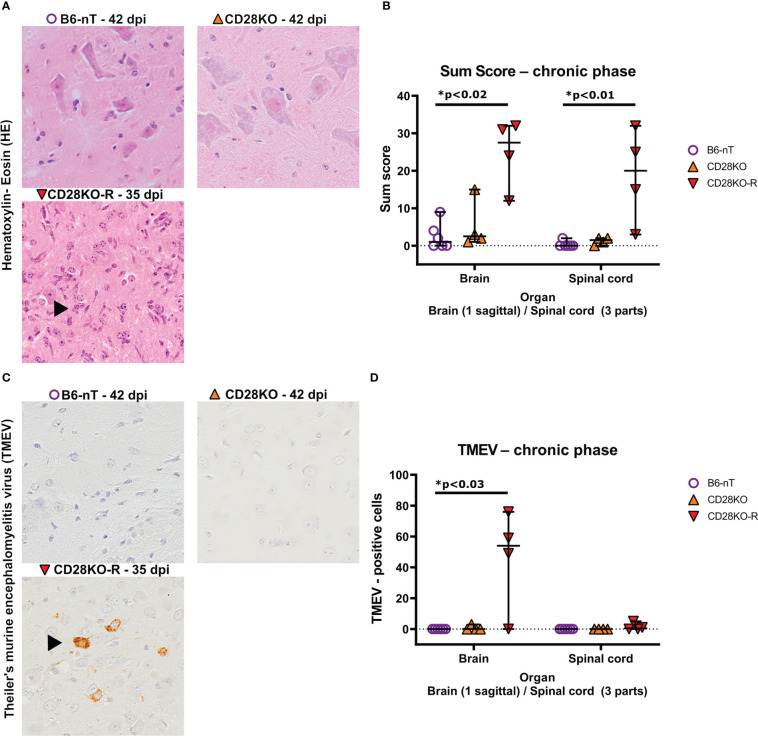
**(A, B)** Semi-quantitative scoring of brain and spinal cord on hematoxylin-eosin (HE) stained sections in the chronic phase at 33, 35 or 42 days post infection (dpi), respectively. Theiler’s murine encephalomyelitis virus (TMEV)-infected CD28-knockout mice with clinical signs in the chronic phase, termed ‘responder CD28KO’ mice, (CD28KO-R, n=4) were euthanized at 33 (n=1), 35 (n=1) and 42 dpi (n=2) and compared to CD28-knockout mice without clinical signs (CD28KO, n=4) and control mice without knockout (B6-nT, n= 6) that were sacrificed on 42 dpi. **(A)** Representative pictures of lumbar spinal cord of TMEV-infected mice showing lack of inflammatory changes in B6-nT (upper left) and CD28KO mice without clinical signs (upper right) in contrast to ongoing inflammation (arrowhead) in the spinal cord of a CD28KO-R mouse at 35 dpi (lower left). **(B)** Semi-quantitative scores representing the total sum obtained by added scores of 10 different brain- or 6 spinal cord regions, respectively, showed significantly increased scores in the brain and spinal cord of CD28KO-R mice compared to B6-nT controls in the chronic phase of TMEV-infection. **(C, D)** Immunohistochemical analysis of TMEV-antigen within the brain and spinal cords of mice in the chronic phase of TMEV-infection. **(C)** Representative pictures lack TMEV-antigen in B6-nT control mice (upper left) and CD28KO mice without clinical signs (upper right) in contrast to persisting TMEV-antigen within the cerebral cortex of a CD28KO-R mouse at 35 dpi (lower left). **(D)** Quantitative analysis revealed TMEV-persistence within the brain and spinal cord of CD28KO-R mice. Graphs show scatter plots (one dot per animal) with median (black bar) and 95% confidence intervals (black error bars). Statistically significant p-values are depicted within the graphs (Kruskal-Wallis test followed by Dunn-Bonferroni *post-hoc* test and significance level correction for multiple testing).

Histopathological examination at 42 dpi revealed that B6-nT control animals without knockout (**Figure 5A**, upper left) as well as conventional CD28KO mice without gait abnormalities (**Figure 5A**, upper right) showed no, or only minor inflammation characterized by single parenchymal and perivascular infiltrations of immune cells within the brain and spinal cord (**Figure 5B**). In contrast, inflammation was still visible within the spinal cord of the CD28KO-R mice and most prominent in the lumbar segment (**Figures 5A, B**). The B6-nT controls (**Figure 5C**, upper left) and clinically unaffected CD28KO mice (**Figure 5C**, upper right),except for one mouse with very low titer, were able to eliminate TMEV from the brain and spinal cord (**Figure 5D**) at 42 dpi. Conversely, TMEV-antigen was still detectable within the brain (**Figure 5C**, lower left) of 3 and spinal cords of 2 out of 4 CD28KO-R mice, respectively (**Figure 5D**).

**Figure 6:**
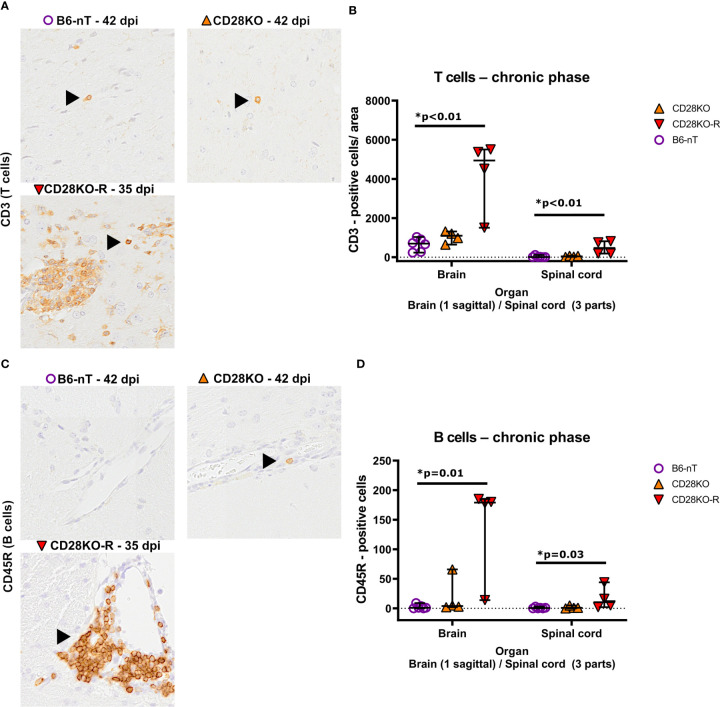
**(A, B)** Immunohistochemical analysis of CD3-positive T cell infiltration into the brain and spinal cord of Theiler’s murine encephalomyelitis virus (TMEV)-infected mice in the chronic phase at 33, 35 and 42 days post infection (dpi), respectively. TMEV-infected conventional CD28-knockout mice with clinical signs in the chronic phase, termed ‘responder CD28KO’ mice, (CD28KO-R, n=4) were euthanized at 33 (n=1), 35 (n=1) and 42 dpi (n=2) and compared to CD28-knockout mice without clinical signs (CD28KO, n=4) and control mice without knockout (B6-nT, n= 6) that were sacrificed on 42 dpi. **(A)** Representative pictures showing single CD3-positive T cells within the hypothalamus (B6-nT, upper left)) and thalamus (CD28KO, upper right) in contrast to increased T cell infiltration into the thalamus of a TMEV-infected CD28KO-R mouse (lower left) at 35 and 42 dpi, respectively. **(B)** Quantitative evaluation of T cells per area (0.5 cm² brain, 0.07cm² spinal cord) shows, compared to B6-nT controls at 42 dpi, a significantly increased T cell infiltration into the brain and spinal cord of CD28KO-R mice at 33, 35 and 42 dpi, respectively. **(C-D)** Immunohistochemical analysis of CD45R-positive B cell infiltration into the brain and spinal cord of TMEV-infected mice in the chronic phase at 33, 35 and 42 dpi, respectively. **(C)** Representative pictures of CD45R-positive B cells (arrowheads) within the hypothalamus, showing no B cells in a B6-nT (upper left) or single perivascular B cells in a CD28KO mouse (upper right), in contrast to increased perivascular infiltration in a CD28KO-R mouse at 35 dpi (lower left). **(B)** Quantitative analysis of B cell infiltration shows significantly increased numbers of B cells within the brain and spinal cord of CD28KO-R mice at 33, 35 and 42 dpi, compared to B6-nT controls at 42 dpi. Graphs show scatter plots (one dot per animal) with median (black bar) and 95% confidence intervals (black error bars). Statistically significant p-values are depicted within the graphs (Kruskal-Wallis test followed by Dunn-Bonferroni *post-hoc* test and significance level correction for multiple testing).

The low sum score of the CNS of B6-nT control animals and non-responder CD28KO mice, were affirmed by low numbers of infiltrating T cells (**Figures 6A, B**) and B cells (**Figures 6C, D**). This was in contrast to the ongoing T cell (**Figures 6A, B**) and B cell (**Figures 6C, D**) infiltration in CD28KO-R mice.

**Figure 7:**
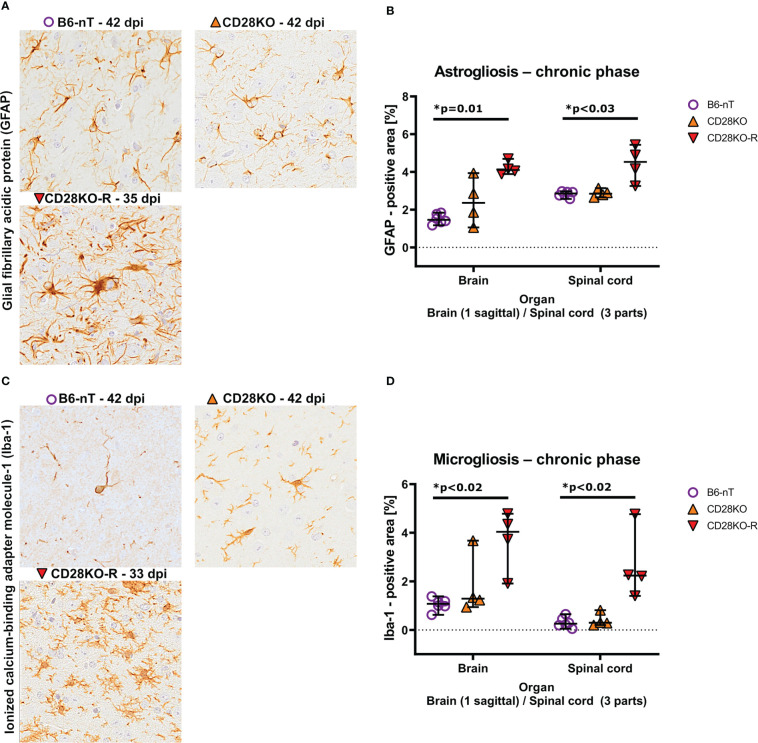
**(A, B)** Immunohistochemical analysis of glial fibrillary acidic protein (GFAP)-positive astroglia (astrogliosis) within the brain and spinal cord of Theiler’s murine encephalomyelitis virus (TMEV)-infected mice in the chronic phase at 33, 35 and 42 days post infection (dpi), respectively. TMEV-infected conventional CD28-knockout mice with clinical signs in the chronic phase, termed ‘responder CD28KO’ mice, (CD28KO-R, n=4) were euthanized at 33 (n=1), 35 (n=1) and 42 dpi (n=2) and compared to CD28-knockout mice without clinical signs (CD28KO, n=4) and control mice without knockout (B6-nT, n= 6) that were sacrificed on 42 dpi. **(A)** Representative pictures of GFAP-positive astroglia (arrowheads) within the thalamus of TMEV-infected mice, showing moderate numbers of astroglia with few processes in the brains of B6-nT (upper left) and CD28KO mice (upper right) at 42 dpi in contrast to increased cell size and number of processes in a CD28KO-R mouse (lower left) at 35 dpi. **(B)** Quantitative analysis of GFAP-positive area revealed significantly increased astrogliosis within the brain and spinal cord of CD28KO-R mice compared to B6-nT controls in the chronic phase. **(C, D)** Analysis of activated, ionized calcium-binding adapter molecule-1 (Iba-1)-positive microglia within the brain and spinal cord of TMEV-infected mice at 33, 35 and 42 dpi, respectively. **(C)** Representative pictures of Iba-1-psotive microglia/macrophages (arrowheads) within the thalamus of TMEV-infected mice at 33 and 42 dpi showing low cell numbers and few processes in B6-nT (upper left) and CD28KO mice (upper right) at 42 dpi, in contrast to increased ramification of microglia within a CD28KO-R mouse (lower left) at 33 dpi. **(D)** Quantitative analysis of Iba-1-positive area revealed significantly increased microgliosis within the brain and spinal cord of CD28KO-R mice compared to B6-nT controls. Graphs show scatter plots (one dot per animal) with median (black bar) and 95% confidence intervals (black error bars). Statistically significant p-values are depicted within the graphs (Kruskal-Wallis test followed by Dunn-Bonferroni *post-hoc* test and significance level correction for multiple testing).

Compared to the B6-nT controls (**Figures 7A–D**, upper left) the astroglial and microglial response in the brain was mildly but not significantly elevated in CD28KO mice without clinic (**Figures 7A–D**, upper right). CD28KO-R mice showed significantly increased density of astrocytes (astrogliosis) within the brain and spinal cord (**Figures 7A, B**) as well as a high density of hypertrophic/hyper-ramified microglia (**Figures 7C, D**). These data indicate an ineffective and delayed immune response against TMEV-infection in clinically affected CD28KO-R mice.

**Figure 8:**
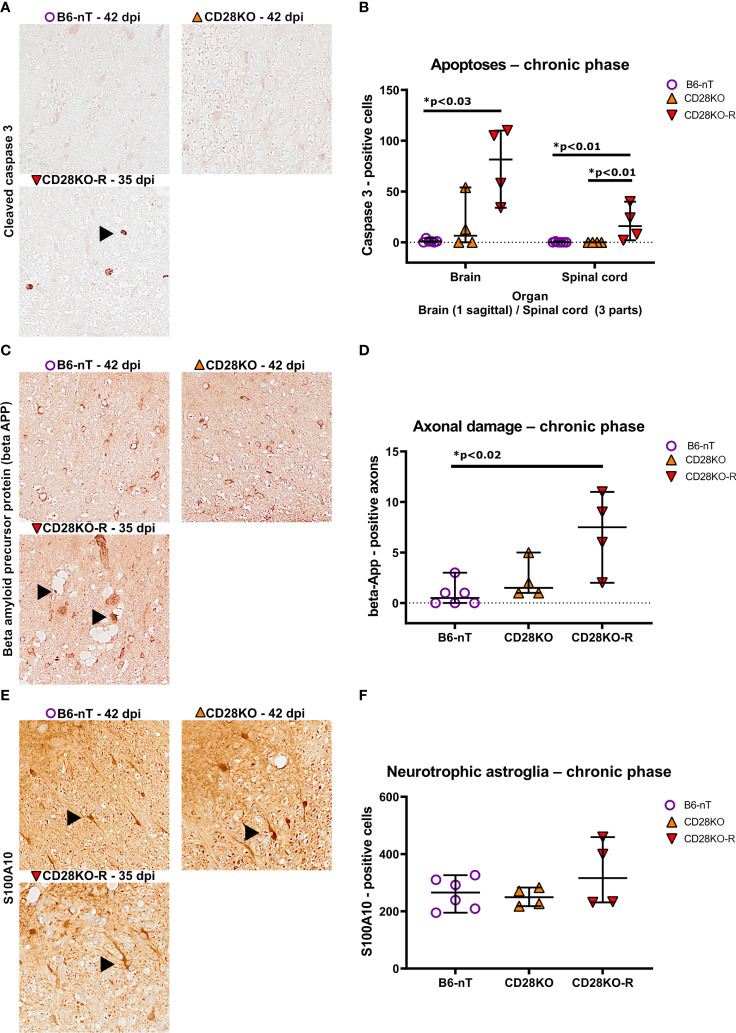
**(A, B)** Immunohistochemical analysis of cleaved caspase 3-positive, apoptotic cells within the brain and spinal cord of Theiler’s murine encephalomyelitis virus (TMEV)-infected mice in the chronic phase at 33, 35 and 42 days post infection (dpi), respectively. TMEV-infected conventional CD28-knockout mice with clinical signs in the chronic phase termed ‘responder CD28KO’ mice (CD28KO-R, n=4), were euthanized at 33 (n=1), 35 (n=1) and 42 dpi (n=2) and compared to CD28-knockout mice without clinical signs (CD28KO, n=4) and control mice without knockout (B6-nT, n= 6) that were sacrificed on 42 dpi. **(A)** Representative pictures of the lumbar spinal cord show no apoptoses in B6-nT (upper left) and CD28KO mice (upper right) at 42 dpi in contrast to low numbers of apoptotic cells (arrowhead) in a CD28KO-R mouse (lower left) at 35 dpi. **(B)** Quantitative analysis of cleaved caspase 3-positive cells revealed significantly increased numbers of apoptoses within the brain and spinal cord of CD28KO-R mice compared to B6-nT controls. **(C, D)** Immunohistochemical analysis of beta amyloid precursor protein (beta-APP)-positive, damaged axons within the spinal cord of TMEV-infected mice at 33, 35 and 42 dpi. **(C)** Representative pictures show no damaged axons in B6-nT (upper left) and CD28KO mice (upper right) at 42 dpi, in contrast to the CD28KO-R mouse (lower left) showing small and enlarged beta-APP-positive axons (arrowheads) within dilated myelin sheets in the lumbar spinal cord at 35 dpi. **(D)** Quantitative analysis of beta-APP-positive axons revealed significantly increased axonal damage within the spinal cord of CD28KO-R mice compared to B6-nT controls. **(E, F)** Immunohistochemical analysis of S100A10-positive, neurotrophic astroglia within the spinal cord of TMEV-infected mice in the chronic phase at 33, 35 and 42 dpi, respectively. **(E)** Representative pictures show S100A10-positive astroglia (arrowheads) within the spinal cord of all TMEV-infected mice in the chronic phase of TMEV-infection. **(F)** Quantitative analysis revealed no significant difference between the groups regarding the number of neurotrophic astroglia in the chronic phase. Graphs show scatter plots (one dot per animal) with median (black bar) and 95% confidence intervals (black error bars). Statistically significant p-values are depicted within the graphs (Kruskal-Wallis test followed by Dunn-Bonferroni *post-hoc* test and significance level correction for multiple.

Concomitant to the chronic TMEV-infection and ongoing inflammation within the CNS of CD28KO-R mice, the responders also show increased numbers of apoptotic cells (**Figures 8A, B**) and damaged axons within the spinal cord (**Figures 8C, D**). The number of neurotrophic S100A10-positive astrocytes in the spinal cord was not affected (**Figures 8E, F**). Prominent loss of myelin was not seen in luxol-fast-blue (LFB)-stained sections of the spinal cord in any of the mice (data not shown). When following the remaining B6-nT control animals (n=7) and conventional CD28KO animals (n=4) up to 147 dpi, no development of clinical signs or histopathological abnormalities were observed (data not shown).

In summary, in the chronic phase after TMEV-infection, the deletion of CD28 results in variable outcome. Control mice (as expected) and two third of conventional CD28KO mice are able to resolve the virus infection without development of clinical symptoms and histopathological abnormalities. In contrast, one third of CD28KO mice are susceptible for a chronic TMEV-infection with an inefficient inflammatory response and concomitant loss of lower motor neurons within inflamed spinal cord regions as well as increased apoptosis and axonal damage.

## 4 Discussion

The deletion of the co-stimulatory molecule CD28 has considerable impact in the TMEV-model at different time points after virus inoculation as determined by clinical evaluation, functional testing and pathohistological analysis. In the early phase at 14 dpi, both conventional and conditional deletion of CD28 resulted in impaired viral clearance with spread of TMEV within the brain and into the spinal cord, the latter being a rather uncommon finding in C57BL/6 mice. Furthermore, despite more abundant T cell infiltration, the microglial activation was reduced in both mouse strains, which correlated with the inability for the clearance of the virus. The chronic phase of TMEV-infection was only investigated in conventional CD28KO mice, due to animal welfare reasons. Two third of the animals did not develop clinical symptoms and showed no histopathological abnormalities when investigated at 42 or 147 dpi, pointing to a rather delayed then abolished immune response and eventual clearance of the virus. However, one third of the CD28KO mice developed clinical symptoms with loss of motor function in the chronic phase (CD28KO-R), indicating that these mice could not clear the virus with fatal consequences. The clinical signs seen between 21-42 dpi were associated with virus persistence and increased inflammation within the CNS of CD28KO-R mice, characterized by increased T- and B cell infiltration, microglial activation and astrogliosis at 33-42 dpi.

In summary, loss of CD28 in any case resulted in an impaired anti-viral immune response reflected by abnormal histopathology and accompanied by the development of clinical symptoms already in the early phase in the case of conditional deletion of CD28 and in one third of the mice in the case of permanent deletion of CD28 in the chronic phase of the disease.

### 4.1 CD28 and controlling a virus infection in the CNS – immune cell homeostasis

The infection of C57BL/6 mice with low virulent TMEV is known to cause acute and chronic seizures and is thus used as a model for human temporal lobe epilepsy (37). However, the occurrence and frequency of seizures depends on the virus strain (38). About 50%-75% of mice intracerebrally infected with the Daniel’s strain of TMEV (TMEV-DA) develop seizures, depending on the applied detection method and initial virus dose (39–41). For the TMEV-BeAn-strain the percentage differs between 0-40% (39, 41). It was seen that seizure development does not correlate with neuropathological changes (39). In the present experiment the variant BeAn-1-TiHo was used, which is not suspected to cause seizures in C57BL/6 mice (41). Concomitantly, no apparent seizures were observed after TMEV-BeAn-infection in the present study. However, it cannot be excluded that seizures might have occurred apart from the general examinations, since no specific scoring (Racine score) or continuous monitoring *via* video-electroencephalogram (VEEG) were applied.

One of the hallmarks after CD28 loss – both conventional and conditional - is the unexpected virus spread within the brain as well as into the spinal cord of mice with a C57BL/6 background (**Figures 2C, D**). In general, C57BL/6 mice are considered to be resistant to a chronic TMEV-infection and are able to eliminate the virus within 2 weeks after intracranial infection (9). Although it was previously already shown that the tamoxifen application is associated with delayed virus elimination (34), the tamoxifen treated mice without CD28 knockout (B6-Tam) did not show virus spread into the spinal cord, or elevated clinical scores. In both CD28-knockout strains, microglial activation and change of morphology as reaction to the acute TMEV-infection in the brain were reduced.

Brain-resident microglia and infiltrating monocytes as part of the innate immune response play a pivotal role in the acute, as well as in the chronic phase of a TMEV-infection (42). On the one hand, these cell types contribute to local damage, scarring and development of seizures in C57BL/6 mice (42). Further, microglia are susceptible for TMEV-infection and the major host cells during the chronic TMEV-BeAn infection as seen in SJL mice (43). On the other hand, a clearing of TMEV from the CNS is impossible for C57BL/6 mice after depletion of microglia (44). Similarly, the presented data indicate a link between reduced microglial activation (**Figures 3E, F**) and delayed virus elimination and virus spread within the CNS in mice lacking CD28 (**Figures 2C, D**). The molecular background of this correlation will be subject of further studies.

For infiltration of cells of the adaptive immune response (T- and B cells), the situation is different. As described in other studies on the immune system (45), conventional TMEV-infected CD28KO mice had similar levels of infiltrating T- and B cells into the brain and spinal cord as their B6-nT controls. Conversely, Cre-Tam mice with an induced knockout showed increased T-cell infiltration and almost a total lack of B cell infiltration into the TMEV-infected brain. Furthermore, it was shown that the tamoxifen application alone (B6-Tam) has a similar effect, but to a lesser extent. Thus, it seems that the tamoxifen application and the delayed anti-viral immune response is the main reason for the changed T- and B-cell infiltration, and that the actual deletion of CD28 is somehow reinforcing these effects, whereas the pure lack of CD28 from the beginning has no influence of T- and B cell infiltration. Based on these data, it also seems that T- and B cell infiltration is uncoupled from microglial activation after TMEV-infection and also from the development of neurological symptoms.

CD28 is important during the antigen presentation from myeloid cells, for instance dendritic cells (DC), but also decisive in the communication between T- and B cells (46). Antigens binding to the B cell-receptor (BCR) are processed and presented on MHC-II with concomitant expression of CD80/CD86 (B7-1/B7-2) as ligands for CD28 on the T cell (46, 47). Activated T cells secrete interleukins to further stimulate B cells, however, the interaction of CD28 with the B7 molecules itself poses a direct co-stimulatory signal for B cells (48). The presented data show, that the activation and expansion of B cells is almost completely aborted in mice with induced CD28 knockout, which is likely due to a loss of communication between the B- and T cells as well as loss of CD28/B7 mediated co-stimulation of B cells (48). The similarity in T- and B cell reaction of mice with conventional CD28 knockout in this study indicates an alternative route of activation in these mice. Although CD28 is a main molecule for the activation of naïve T cells, it is by far not the only one (49). Upregulation of other co-stimulatory molecules may have compensated the lack of CD28-signaling in the thymus of CD28KO mice with conventional CD28-knockout (45, 49, 50).

Tamoxifen as a selective estrogen receptor modulator (SERM) is known to influence the immune response under therapeutic as well as in experimental conditions (4, 34, 51–53). Although tamoxifen is designed to act on human estrogen receptors (hER) it also influences murine immune cells and has estrogen-like (agonistic) as well as antagonistic properties, depending on the expression of ER-subtypes (4, 34, 51–57). Since tamoxifen is a SERM and not a specific ER-inhibitor, and it also modulates cell responses through estrogen-independent mechanisms, reported effects on the immune response on a cellular level are divergent and highly depend on the applied method (51, 58). In the CNS, tamoxifen has mostly neuroprotective and anti-inflammatory properties, increases proliferation and differentiation of oligodendrocyte progenitor cells (OPC), decreases production of pro-inflammatory cytokines like TNF-alpha, IL-12 and IL-1 by micro- and astroglia, as well as reduces astrogliosis under non-infectious conditions (59, 60). Under infectious conditions, these effects might affect the initial immune response to be less efficient, resulting in a delayed virus elimination as seen in tamoxifen treated animals in the present study. The ongoing presence of TMEV-antigen is however suspected to stimulate the immune response in these animals, possibly covering an initially anti-inflammatory effect until 14 dpi. Further, tamoxifen is known to negatively influence the activation and maturation of B cells (54). The B cell response after a virus infection is dependent on factors of the virus itself, such as pathogenicity and cell tropism, as well as the accessibility and presentation of virus peptides and a pro- or anti-inflammatory microenvironment (61, 62). Although ER-β-mediated effects of tamoxifen are suspected to have negative effects on B cells (63), the molecular mechanism behind the reduced B cell response remains unclear for the present study and demands further investigation.

Despite differences in T- and B cell infiltration into the CNS of TMEV-infected mice with conventional and induced CD28-knockout, the virus elimination in these mice was equally impaired. The weak microglial activation in the early phase of TMEV-infection in both types of CD28-knockout mice indicates a disruption of an activation pathway, which could not be quickly overcome by other routes of co-stimulation and is somewhat independent of the B- and T cell infiltration. A comparable stimulatory function of B7-signaling on microglia (64), similar to B cells (48), might be a factor that should be addressed in future studies.

Since the cre-expression of these mice is not controlled by a cell-specific promotor, but rather expressed in all cells after tamoxifen application, a CD28 knockout in all cells is expected in the Cre-Tam mice. The variable significant difference in T- and B cell infiltrations of Cre-Tam mice compared to the TMEV-infected mice without CD28-knockout, as well as the decreased microglial reaction during the acute phase of TMEV-infection supports this assumption of a CD28-knockoutin the infiltrating immune cells.

The role of a CD28-knockout for CD4^+^ and CD8^+^ T cells separately represent an interesting topic for future studies in this model. The outcome and special contributions of MHC-I molecule H-2D^b^ (65, 66), the early anti-viral CD8^+^T cell response to a TMEV-infection, the reaction of CD4^+^ T helper and effector cells as well as regulatory T cells (Tregs) (67, 68) could not be differentiated in the present study, utilizing a generalized CD28-knockout. A cell specific promotor for the cre-expression might aid to shed light on the contribution of different immune cell subsets to the observed neuropathologies in the present study (69). It would further be of interest to implement this model in mice on a susceptible SJL-background to investigate the effect of a CD28-knockout on demyelination (TMEV-IDD) (1).

### 4.2 Chronic infection of mice with conventional CD28-knockout

Due to the acute clinical and pathomorphological impairments of conditional CD28-knockout at 14 dpi, this mouse strain could not be included in long-term studies. Therefore, the chronic TMEV-infection was only investigated in mice with conventional CD28-knockout (CD28KO).

A surprising finding here was the heterogeneity in the long-term clinical outcome of the intracerebral TMEV-infection in CD28KO mice. Two thirds of CD28KO mice were, just like the B6-nT control animals, clinically unaffected until 42 dpi or even 147 dpi (data not shown). 33% of the animals showed progressive impairments of motor function (CD28KO-R, ‘responders’), starting around 21-33 dpi, leading up to hind-limb paresis in 2 of 4 affected animals.

Analysis of body weight, clinical score and RotaRod-performance test of the CD28KO-R did not reveal changes in the acute phase of TMEV-infection that would signal that these animals would develop clinical signs during the chronic phase. From 14 dpi until 42 dpi, the median RotaRod-performance of CD28KO-R mice is reduced compared to the other groups, followed by first clinical signs in some mice at 21 dpi and a lower median body weight from 28 until 42 dpi (**Supplementary Figure S3**).

Histologically, the responder-mice (CD28KO-R) were affected by ongoing virus spread and CNS-inflammation, indicating that the virus could not be cleared due to an inefficient immune response. However, in contrast to susceptible SJL mice (4), the myelitis of CD28KO-R mice was not associated with prominent demyelination, but inflammation and possible damage of lower motor neurons in the ventral horns. Thus, autoimmunity did not develop in the absence of CD28 in C57BL/6 mice, even when the virus could not be cleared similar to SJL mice. The marked difference to the early phase for the CD28-knockout mice was the increased microglial and astroglial activation at 33-42 dpi, combined with high numbers of infiltrating lymphocytes. However, despite this inflammatory response and microglial activation, the CD28KO-R mice were not able to eliminate the virus. It would be interesting to investigate the trigger(s) that differentiate mice able versus unable to clear the virus on the long run.

CD28 signaling is not only important for T cell activation and priming but also plays an important role for effector T cells (70). In CD28^flox/flox^ Ox40^cre/+^ mice where CD28 expression is lost after T cell priming following an influenza A infection, it was shown that T follicular helper cells (Tfh) need CD28 for survival, and T helper (Th1) require CD28-signaling for expansion after activation (70). These findings corroborate studies that showed a decreased germinal center formation and isotype switching in CD28-deficient C57BL/6 mice (20, 71). Conversely, it was shown that stimulation of the CD28-receptor on human and murine Th1 cells induces IFN-γ secretion (72). In microglia and astrocytes, IFN-γ and other Th1 effectors regulate gene expression associated with neuroinflammation (73), indicating that a loss of CD28-sinaling might have an anti-inflammatory effect. Further, the interaction of CD28 with the B7-ligands not only stimulates cytokine production in T cells but also triggers auto- and paracrine IL-6 and IFN-γ-secretion by dendritic cells (DC) (74). This bidirectional signaling of CD28 and B7 molecules has yet to be shown for microglia, as a possible factor for initially decreased microgliosis in TMEV-infected, CD28-knockout mice. However, in the present study it was seen, that a chronic TMEV-infection of CD28KO mice is associated with ongoing neuroinflammation, characterized by T- and B cell infiltration as well as astro- and microgliosis. In the early phase of TMEV-infection, the T- and B cell response of CD28KO mice does not differ significantly from the B6-nT controls. This indicates a CD28-independent activation pathway in lymphocytes of conventional CD28KO mice, and might indicate a disturbed interaction of lymphocytes and microglia in the early phase of TMEV-infection, as reported for DCs (74). The glial reaction in the late phase of TMEV-infection is likely reflective of the pro-inflammatory microenvironment, sustained by activated T and B cells in response to virus persistence.

Besides CD28, T cells own a large variety of co-stimulatory and co-inhibitory receptors whose expression depends on the cell’s environment and activation status (49). Hence, despite their narrow genetic diversity, also inbred mice own individual expression panels of TCRs and co-receptors (20). In absence of CD28, individual expression of co-receptors could explain the heterogeneity in virus persistence and clinic seen in mice with a lack of CD28 (75).

## 5 Summary and conclusions

In summary, the absence of CD28 in T cells severely impacts the immune response against Theiler`s murine encephalomyelitis virus (TMEV)-infection, but significant differences are also seen depending on the time point of the deletion during the development of the immune system. CD28KO mice with a conventional CD28-knockout appear to develop early and individual compensatory mechanisms to overcome the lack of CD28 co-stimulation in the thymus as well as peripheral T- and B cells. Therefore, they do not develop clinical signs in the early phase of the infection. However, nevertheless these alternative pathways are inefficient under infectious conditions as indicated by delayed virus clearance accompanied by histopathological abnormalities in the early phase and the inability to finally clear the virus in one third of the mice in the chronic phase. This phenotype is much more pronounced in Cre-Tam mice with a tamoxifen-induced, conditional knockout of CD28. In these mice, viral spread within the brain and into the spinal cord was also observed in the early phase of the disease. Additionally, they show reactive T cell infiltration into the TMEV-infected brain, and develop clinical signs accompanied by a significantly reduced body weight already in this stage. Therefore, the immune system obviously has more problems to deal with virus infections when CD28 is suddenly lost without the possibility that adaptive mechanisms take place. However, the persistent virus infection within the CNS in these CD28-deficient C57BL/6 mice does not lead to autoimmunity as seen in susceptible mouse strains such as SJL, leading to the conclusion that other factors besides virus persistence may contribute to this phenotype.

## Data availability statement

The original contributions presented in the study are included in the article/**Supplementary Material**. Further inquiries can be directed to the corresponding author.

## Ethics statement

The animal study was reviewed and approved by Lower Saxony State Office for Consumer Protection and Food Safety (LAVES), 26029 Oldenburg, Germany.

## Author contributions

KH: planning and execution of animal experiment, laboratory work, analysis, manuscript preparation, figures, statistics; FL: planning of experiment, generation of genetically engineered mice, writing and revision of the manuscript; EL: statistics, manuscript editing; AF: planning of experiment, revision of the manuscript; WB: planning of experiment, revision of the manuscript, corresponding author. All authors contributed to the article and approved the submitted version. 
