# Supplementary material for: CD28-signaling can be partially compensated in CD28-knockout mice but is essential for virus elimination in a murine model of multiple sclerosis
Source: Front Immunol. 2023 Apr 5;14:1105432. doi: 10.3389/fimmu.2023.1105432 (PMC10113529; doi:10.3389/fimmu.2023.1105432)
Supplement: Supplementary file 1 [file DataSheet_1.docx]

Supplementary Material

# Supplementary Figures


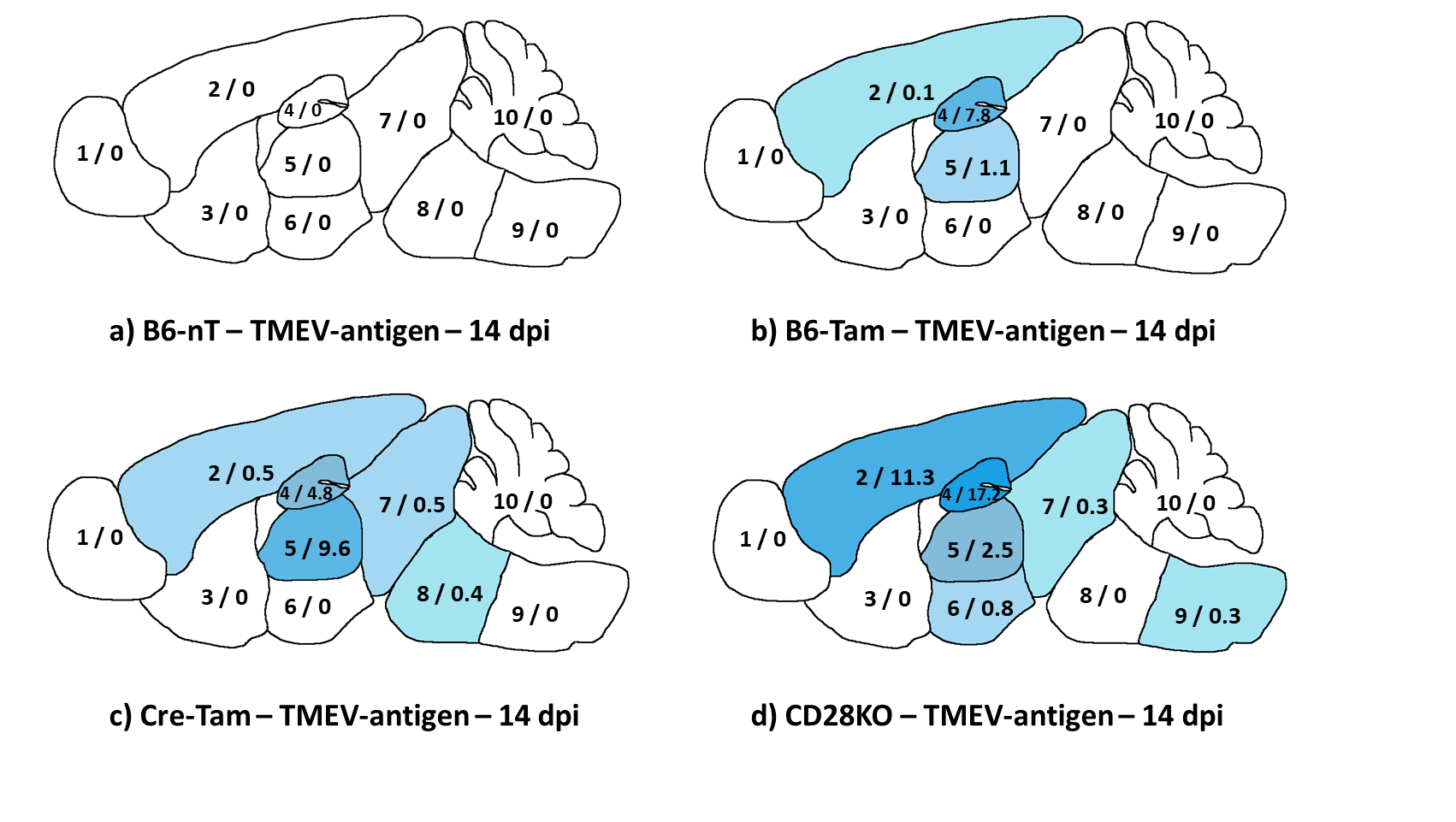


**Figure S1:** Scheme of separately evaluated brain regions of Theiler’s murine encephalitis virus (TMEV) infected mice at 14 days post infection (dpi). Sagittal brain sections were divided into 10 areas: 1/ olfactory bulb, 2/ cerebral cortex, 3/ forebrain, 4/ hippocampus, 5/ thalamus, 6/ hypothalamus, 7/ midbrain, 8/ pons, 9/ medulla oblongata and 10/ cerebellum. Depicted are the mean numbers of TMEV-positive cells in the respective regions within the different animal groups of mice without tamoxifen application and no knockout (B6-nT), mice with tamoxifen application and no knockout (B6-Tam), mice with conditional, tamoxifen-induced knockout of CD28 (Cre-Tam) and mice with conventional innate CD28-knockout (CD28KO) 14 dpi. **a)** B6-nT mice were able to eliminate the virus from their brain within 14 days. **b)** B6-Tam mice showed low numbers of TMEV-antigen positive cells within their cerebral cortex (mean= 0.1) and thalamus (mean= 1.1) as well as higher numbers within the hippocampus (mean= 7.8). **c)** Cre-Tam mice showed low numbers of TMEV-antigen positive cells within their cerebral cortex (mean= 0.5), midbrain (mean= 0.5) and pons (mean= 0.4) as well as moderate numbers within the hippocampus (mean= 4.8) and thalamus (mean= 9.6). **d)** CD28KO mice showed low numbers of TMEV-antigen positive cells within the hypothalamus (mean= 0.8), midbrain (mean= 0.3) and medulla oblongata (mean= 0.3), moderate numbers within the thalamus (mean= 2.5) and high numbers within the cerebral cortex (mean= 11.3) and hippocampus (mean=17.2). **a-b)** In mice without CD28-knockout 0 (B6-nT) to 3 of 10 areas (B6-Tam) contain TMEV-antigen positive cells at 14 dpi. **c-d)** Mice with knockout of CD28 show TMEV-antigen in 5 (Cre-Tam) to 6 of 10 regions (CD28KO) at 14 dpi.


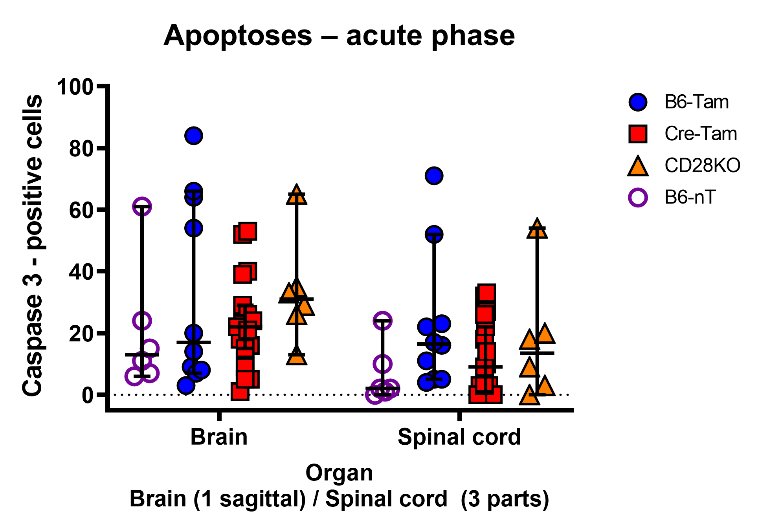

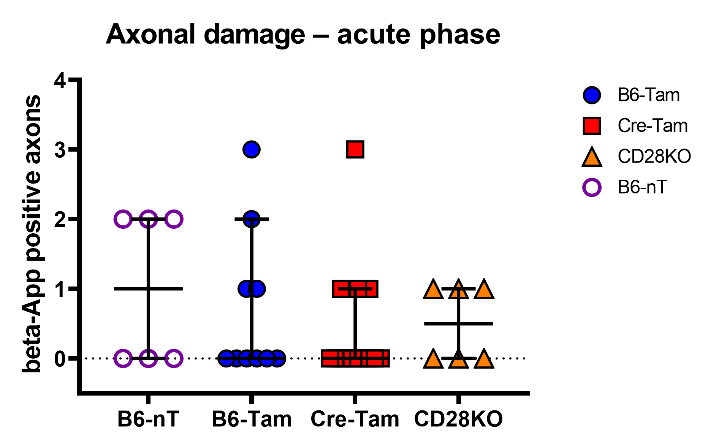


**Figure S2:** Number of apoptoses (Cleaved caspase 3 positive cells) and damaged axons (Beta amyloid precursor protein [beta APP] positive axons) in the brain and spinal cord of Theiler’s murine encephalitis virus (TMEV) infected mice at 14 days post infection (dpi). There are no significant differences in apoptoses in brain and spinal cord or axonal damage within the spinal cord at 14 dpi. Mice without (B6-nT, purple circles) and with tamoxifen application (B6-Tam, blue dots) and no CD28-knockout, as well as mice with conditional, tamoxifen-induced (Cre-Tam, red squares) and conventional innate CD28-knockout (CD28KO, orange triangles) show similar numbers of apoptotic cells within the central nervous system (CNS) and no elevation of axonal damage at 14 dpi. Graphs show scatter plots (one dot per animal) with median (black bar) and 95% confidence intervals (black error bars).


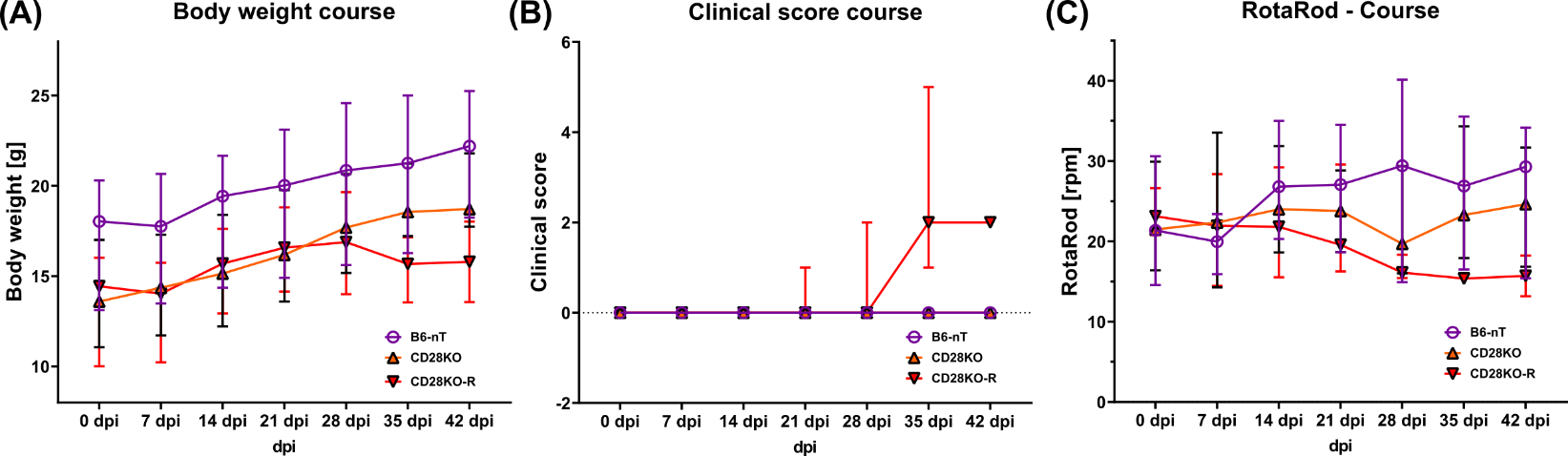


**Figure S3:** (A-C) Course of clinical data from B6-nT controls (purple), CD28-knockout mice without clinical signs (CD28KO, orange), and CD28-knockout mice with clinical disease (CD28KO-R, red). (A) At 28, 35 and 42 days post infection (dpi), the median body weight of CD28KO-R mice is lower compared to unaffected CD28KO mice. (B) First clinical signs in CD28KO-R mice are shown at 21 dpi. (C) From 14 dpi until 42 dpi, the median RotaRod-performance of CD28KO-R mice is reduced compared to the other groups. Graphs show median values of the groups and standard deviation (error bars).
